# Gene Therapy for Progressive Familial Intrahepatic Cholestasis: Current Progress and Future Prospects

**DOI:** 10.3390/ijms22010273

**Published:** 2020-12-29

**Authors:** Piter J. Bosma, Marius Wits, Ronald P. J. Oude-Elferink

**Affiliations:** Tytgat Institute for Liver and Intestinal Research and Department of Gastroenterology and Hepatology, AGEM, Amsterdam UMC, University of Amsterdam, 1105 BK Amsterdam, The Netherlands; marius_wits@hotmail.com (M.W.); r.p.oude-elferink@amsterdamumc.nl (R.P.J.O.-E.)

**Keywords:** gene therapy, AAV, PFIC

## Abstract

Progressive Familial Intrahepatic Cholestasis (PFIC) are inherited severe liver disorders presenting early in life, with high serum bile salt and bilirubin levels. Six types have been reported, two of these are caused by deficiency of an ABC transporter; ABCB11 (bile salt export pump) in type 2; ABCB4 (phosphatidylcholine floppase) in type 3. In addition, ABCB11 function is affected in 3 other types of PFIC. A lack of effective treatment makes a liver transplantation necessary in most patients. In view of long-term adverse effects, for instance due to life-long immune suppression needed to prevent organ rejection, gene therapy could be a preferable approach, as supported by proof of concept in animal models for PFIC3. This review discusses the feasibility of gene therapy as an alternative for liver transplantation for all forms of PFIC based on their pathological mechanism. **Conclusion:** Using presently available gene therapy vectors, major hurdles need to be overcome to make gene therapy for all types of PFIC a reality.

## 1. Introduction

### 1.1. Clinical Challenge

Progressive Familial Intrahepatic Cholestasis (PFIC) is a heterogenic group of recessively inherited severe liver disorders [1,2]. All types present during infancy or childhood with increased serum bile salts and bilirubin, and pruritus, having a major impact on health-related quality of life. All forms of PFIC are rare autosomal recessive diseases and deficiency of the bile acid export pump (BSEP) activity impairing bile salt handling is seen in several forms. Deficiency can be caused by mutations in the *ABCB11* gene encoding BSEP for instance or by loss of BSEP expression due to mutations in the *NR1H4* gene, encoding the Farnesoid X Receptor (FXR) that is essential for BSEP expression [3,4,5,6]. A loss of BSEP presence in the canalicular membrane due to mutations in Myosin VB (*MYO5B*), involved in its intracellular transport, or reduced BSEP activity, due to canalicular membrane integrity and composition, impair bile salt export from the hepatocyte [7,8,9]. In addition to impaired bile salt export, PFIC can be caused by ABCB4 deficiency involved in maintaining phosphatidylcholine presence in bile, needed to moderate the detergent effect of bile salt by forming mixed micelles [10]. Recently, mutations in the Tight Junction Protein 2 encoding gene (*TJP2*) were identified to cause PFIC [11]. Although *TJP2* mutations may affect tight junction integrity, these patients neither suffer from cholestasis nor other cholangiopathies, as would be expected in leakage of bile and has been observed in Claudin deficiency, for instance [1]. All types of PFIC are mono-genetic disorders and most progress to end stage liver disease making a liver transplant inevitable. In view of the adverse effects of this highly invasive treatment, developing novel treatment options, such as liver directed gene therapy, are warranted.

### 1.2. Presentation and Current Treatment

All forms of PFIC present with jaundice and elevated bile acid levels in serum [1]. The disease onset varies between the different types of PFIC. ABCB11/BSEP (PFIC2), TJP2 (PFIC4) and FXR (PFIC5) deficiency, present in the first months after birth and are rapidly progressing. Presentation of ATP8B1 deficiency (PFIC1) is often months after birth and the progression is moderate. Both other forms, ABCB4 (PFIC3) and MYO5B (PFIC6) deficiency, are diagnosed at a later more variable age with moderate to slow progression [1]. Upon diagnosing cholestasis; serum parameters, liver biopsies and genetic analyses are all used to identify the genetic cause of cholestasis. For instance, high gamma GT is only seen in ABCB4 deficiency (PFIC3) [12] while FXR deficiency results in persistent increased Alpha FetoProtein (AFP) levels in serum [6]. Liver biopsies can be used to verify the absence of proteins involved in physiologic bile formation in the canaliculi or other mechanisms like intracellular protein accumulation that cause hepatocyte and cholangiocyte damage. Identification of the cause allows treatment to begin, albeit all current treatments for PFIC, at best, only slow down disease progression. Ursodeoxycholic acid (UDCA) treatment, reducing the hydrophobicity of the bile salt pool, is a first treatment option for all types of PFIC. The efficacy of this treatment depends on the type of mutation, with patients having missense mutations showing a better response compared to those with complete deficiency [5,13]. In patients with missense mutations in the *ATP8B1* or the *ABCB11* gene, that affect protein folding, 4-phenylbutyrate, restored presence of these transporters in the canalicular membrane, albeit at a low level [14,15,16]. Symptoms are mitigated by a reduction in the bile salt pool by biliary diversion or by preventing intestinal uptake by binding of bile acids to cholestyramine [1]. The efficacy and safety of pharmacological inhibition bile acid uptake using inhibitors of the apical sodium dependent bile transporter (ASBT) may be another approach to reduce the bile acid pool [17,18]. Diarrhea is an adverse effect of several of these approaches due to increasing amounts of bile acids reaching the colon. In view of this, FXR activation to lower bile acid synthesis in the hepatocytes maybe an option to overcome this hurdle and in combination with ASBT inhibition could be an effective approach [19,20]. Several of these treatments do relieve the symptoms and slow disease progression but none of these is curative. For the more recently identified genetic causes of PFIC, namely TJP2-, FXR- and MYO5B-deficiency no treatment options have been reported [1].

This lack of effective treatment options results in disease progression in all patients suffering from PFIC. Upon reaching end-stage liver disease, a liver transplantation is needed for patient survival.

Progress in the performance of liver transplantations over the past decades has led to an established, albeit highly invasive, procedure called orthotopic liver transplantation (OLT) [21]. Long-term complications consist of graft loss and an increased risk for infections and cancer development due to immunosuppressive treatment. The early severe liver damage in many patients suffering from PFIC renders liver transplantation during childhood necessary. This procedure has become the standard of care to treat children with end-stage liver disease with a 20 year survival of over 80% [22,23]. Partial liver transplantation improves size matching between grafts and recipients and alleviates the shortage of size matched donor livers without compromising survival [24]. In addition, this also allows living family-related donor transplantations.

For all severely affected patients, a liver transplant is currently the only curative therapy which has been successfully performed in children suffering from PFIC [25]. Additional complications of this major procedure were also reported in these patients. Patients with mutations that completely abolish ATP8B1 function developed hepatic steatosis in the donor liver. This can be prevented when combining the liver transplant with total internal biliary diversion, but extrahepatic symptoms like severe diarrhea persist [2,25,26]. Also in patients with FXR deficiency complications occurred upon transplantation, these patients also developed a fatty liver [1]. In patients with ABCB11 (BSEP) deficiency, the cholestasis symptoms re-occurred after OLT due to an immune response inhibiting BSEP function [27].

These additional complications, the lack of sufficient donor organs, the risk of life-long immune suppression to prevent organ rejection, and an increased risk for infection and cancer, warrant the development of novel treatment options for these devastating diseases. Gene therapy seems one of the potential treatments for these monogenic recessive disorders.

## 2. Gene Therapy

### 2.1. Clinical Applications

Gene addition therapies driven by Adeno Associated Viral (AAV) vectors for recessive monogenic diseases have been developed and, upon showing safety and efficacy, have received market approval, albeit in a limited number. Presently, three AAV-vector based gene therapeutics are available as Glybera, treating Lipoprotein Lipase (LPL)-deficiency, Luxturna [28], treating Retinal Pigment Epithelial (RPE) 65-related retinal dystrophy, and Zolgensma, treating Spinal Muscular Atrophy (SMA) [28,29,30]. The promising safety and efficacy data obtained in small clinical AAV gene therapy studies resulted in their application for registration to obtain market approval. For instance this approach as a treatment for Choroidenemia [31], aromatic l-amino acid decarboxylase deficiency [32], Pompe’s Disease [33,34], Duchenne Muscular Dystrophy (ongoing: NCT03375164), Becker’s Muscular Dystrophy [35], Limb-Girdle Muscular Dystrophy [36], X-linked myotubular myopathy (ongoing: NCT03199469), haemophilia A [37] and haemophilia B [38,39] all show efficacy. However, some safety issues have occurred, especially in clinical trials for diseases requiring systemic high vector doses. Liver toxicity has been reported for AAV doses above 1 × 10^−14^ vg/kg, indicating that this will be a problem for some applications [40]. Gene therapies for ocular diseases have relatively few hurdles to surmount because the eye is easily accessible, highly compartmentalized, immune-privileged [41], and less invasive as the injection occurs non-systemically. SMA treatment with Zolgensma, targeting the motor neurons, urges the use of intrathecal injections and a high vector doses, causing transient liver inflammation [42,43]. Recently, in a trial targeting X-linked myotubular myopathy, there have been two tragic deaths of pediatric patients in the cohort receiving the highest vector dose [44]. Both patients died due to progressive liver dysfunction and subsequently fatal sepsis upon receiving 3 × 10^−14^ vg/kg. Patients treated with a lower vector dose, 1 × 10^−14^ vg/kg, did not experience any severe adverse effects. The liver tropism of AAV vectors, resulting in toxic vector levels in the hepatocytes complicates gene therapy of disorders requiring a high vector dose in the systemic circulation, like for instance all muscular disorders. Within the scope of this review, looking at gene therapy for PFIC, AAV-mediated gene transfer directed to the liver is especially relevant. When treating inherited liver disorders, the AAV tropism for the liver is a major advantage since lower vector doses are needed for therapeutic efficacy. A number of clinical trials to treat Factor VIII or Factor IX deficiency in hemophilia A or B patients have our equitable interest.

The three year follow-up of a dose escalation study to treat haemophilia A, performed by Pasi and colleagues [37], showed long-term efficacy. All 15 adult patients received doses of 6 × 10^−12^ vg/kg up to 6 × 10^−13^ vg/kg of AAV5-h*FVIII* intravenously. No hepatotoxicity was observed, although in some cases elevated Aspartate Transaminase (AST) levels occurred and were successfully treated with glucocorticoids. Factor VIII plasma activity was restored leading to strong decreases in bleeding incidences, and recombinant Factor VIII use. Although after three years, factor VIII plasma levels were still therapeutic, a gradual decrease over time was seen. A longer follow up is needed to learn if factor VIII levels will remain therapeutic or if re-treatment will be needed. Dose escalation studies to treat Factor IX deficiency also proved to be safe and effective since levels up to 40–50% of normal Factor IX activity were reached [39]. Long-term follow up of an older study for Factor IX deficiency showed long-term efficacy [38]. In these patients the AAV8 vector was injected over nine years ago and they still have clinically relevant Factor IX production. To conclude, AAV-mediated liver-directed gene therapy as a one-time treatment proved to be safe and to provide long-term correction in adults, indicating that this treatment strategy is feasible for inherited severe liver disorders.

AAV vectors do not integrate actively in the host genome but persist in the nucleus in episomal form [45,46]. An important advantage of this is the lack of genotoxicity which can for instance result in tumour formation, as seen with integrating retroviral vectors [47,48]. During cell division, these episomes are not copied and not distributed to the daughter cells. This renders AAV vectors less suitable for treatment of liver disorders early after birth. The growth of the liver results in loss of the initial efficacy [49,50]. Hepatocyte proliferation is also induced upon liver damage for instance after a partial hepatectomy [51]. This complicates the application of AAV-mediated gene therapy for disorders resulting in hepatocyte damage, such as Fumarylacetoacetate hydrolase (FAH) deficiency and PFIC. Ongoing safety and efficacy studies for AAV gene therapy are performed in adults targeting diseases that do not cause liver damage. Recent pre-clinical studies do indicate that AAV-mediated gene therapy for PFIC may be feasible.

### 2.2. Pre-clinical AAV-mediated Gene Therapy for PFIC3

ABCB4 deficiency seems the best option to investigate the feasibility of gene therapy as a treatment option for PFIC. This is because of the availability of a suitable animal model and a previous study showing that partial correction by hepatocyte transplantation provides therapeutic correction [52]. The *Mdr*2^−/−^ (*Abcb4*^−/−^) mouse has been demonstrated to be a relevant model for PFIC3 [10,53]. The severity of the pathology depends on the mouse strain, with FVB mice displaying a more severe phenotype compared to the C57Bl/6 mice. Adjustments via the diet, such as cholate supplementation, increase the mouse bile hydrophobicity which worsens the disease phenotype [54]. In a recent study we investigated the feasibility of AAV8-h*ABCB4* mediated correction in adult *Abcb*4^−/−^ mice with a C57Bl/6 background [55]. Upon vector administration, the efficacy was monitored over time until 6 months using dietary cholate administration to mimic the human bile toxicity [54]. This study provided proof of concept by demonstrating long-term correction, as shown by normalization of the liver damage parameters AST, Alanine Transaminase (ALT) and Alkaline Phosphatase (ALP), and the absence of fibrosis. Restoring a sufficient Phosphatidylcholine (PC) content in bile is a pre-requisite for prolonged correction using AAV. In case of insufficient correction, the ongoing hepatocyte proliferation will result in loss of AAV vectors, thereby further reducing PC presence in bile and thus increasing the liver damage. This was demonstrated by Weber et al. with *Abcb4*^−/−^ FVB mice displaying a more severe phenotype [56]. These mice were treated with an AAV vector, consisting of a codon-optimized h*ABCB4* at week 2 after birth and the effect was monitored for 12 weeks. In males, this treatment appeared effective, but in 50% of the females the correction was lost. To overcome this, a second cohort was injected two times, first at week 2 and three weeks later a second dose was given. This protocol provided prolonged correction and is, with regard to severity of the pathological model, more comparable to PFIC3 patients. Both studies do indicate that long-term correction of a disease causing hepatocyte proliferation using non-integrating AAV vectors is feasible but only if the efficacy is sufficient.

In PFIC3 patients, the disease onset is observed in young children and therefore ideally the therapy should be given at an early stage. This complicates the use of a non-integrating vector like AAV. Several studies have shown that the initial correction is lost over time when treating neonatal or juvenile animals [49,50]. Moreover, the induction of proliferation upon (partial) loss of correction will contribute to episomal transgene loss and will accelerate the decrease in therapeutic efficacy. This hurdle can be overcome by strategies that aim for integration of the therapeutic transgene. Siew et al. tested integration of a therapeutic construct, consisting of a liver specific promoter and a codon-optimized human *ABCB4* transgene, flanked by the piggyBac transposase short terminal repeats [57]. Co-administration of this construct with an AAV2/8 vector containing a piggybac expression cassette to juvenile FVB *Abcb*4^−/−^ mice resulted in the integration of the therapeutic construct providing lifelong hABCB4 expression and correction of the disease. Integration is non-random, but transposons do cause integration in close proximity of actively transcribed gene regions, transcription start sites, and open chromatin structures [58,59]. None of the analyzed unique integration regions were linked to genes known to play a role in hepatocellular carcinomas [59]. Nevertheless, this cannot be excluded. Targeting the transgene integration to a safe genomic locus could overcome this safety issue.

An obvious target to ensure integration in an active region in hepatocytes is the Albumin locus. A recent study aimed at Homologous Directed Repair (HDR) mediated integration of a promoterless Factor IX coding region in this locus without using a nuclease [60]. As the episomal construct lacks a promoter, and a self-cleaving protein is added to the transgene, its expression is regulated by the expression of albumin. The absence of a promoter and a nuclease increases safety of this “generide” strategy. The basis of this approach is to deliver a Factor IX encoding transgene flanked by two homologous arms to the albumin gene with complimentary sequences coding for the desired integration region. Using the generide strategy Muro et al. demonstrated that this could be used to correct a metabolic disorder, albeit with a low efficacy [61]. Inducing double strand breaks in this locus will stimulate HDR and integration efficacy. In a follow up study, increased correction was established by combining the generide approach with an AAV-*saCas9* guided to the albumin locus [62]. In all studies neonatal mice were used, modelling the use early after birth, when the liver is actively growing and hepatocytes are proliferating. The latter is a major advantage because during cell division the HDR system is active whereas it is inactive in quiescent cells [63]. In this study, life-long therapeutic correction of the pathology was established with a treatment shortly after birth. Further, no off-target integrations were seen in predicted sites which underlines the safety profile of this gene modulation system [62]. This targeted integration strategy seems a feasible option to treat PFIC shortly after birth.

### 2.3. Prospects of AAV-Mediated Gene Therapy for PFIC1, 2, 4, 5, and 6

The feasibility of in vivo AAV-mediated non-integrating gene therapy has only been reported for PFIC3. In this disease, the detergent activity of bile salts, that causes the pathology, can be neutralized by partial correction of phosphatidylcholine levels in bile [64]. Establishing ABCB4 expression in a sufficient percentage of the hepatocytes will prevent further damage, halt disease progression and stop hepatocyte proliferation, thereby, preventing the loss of episomal AAV vector genomes caused by cell division [55,56]. AAV-mediated in vivo gene therapy using non-integrating strategies seems less feasible for other types of PFIC.

In the case of PFIC1 and 2, their pathophysiology is driven by individual cellular stressors. These stresses are caused by decreased membrane integrity (in PFIC1) [65] and intracellular bile salt accumulation (in PFIC1, and 2) [66]. Therefore, all hepatocytes need to be corrected to stop damage induced hepatocyte proliferation. The high vector dose to ensure transduction of all hepatocytes may cause liver toxicity. The subsequent proliferation to compensate hepatocyte loss will result in loss of non-integrated AAV vector copies causing loss of correction. For both types an integrating gene therapy strategy, preventing the loss of the vector genome upon cell division, seems a pre-requisite for sustained correction. The current state of the art integrating AAV-mediated gene therapy systems, as described above, have a low efficacy but when performed in neonatal mice the percentage of corrected cells can go up to 24% of total hepatocytes [62]. This indicates therapeutic correction in PFIC1, 2 and 3, seems feasible because of the survival benefit of cells with a correctly integrated copy of the relevant gene. In contrast to the episomal AAV vector genomes, these integrated genes are copied during cell division and transferred to both daughter cells. Due to this transfer of the survival benefit to their descendants a limited number of corrected hepatocytes will repopulate the liver as shown for transplanted hepatocytes [52,67].

The pathophysiologic mechanisms causing liver damage in PFIC4, 5 and 6 have been clarified more recently. In PFIC4, the lack of functional TJP2 results in claudin mislocalization leading to paracellular bile leakage [68]. Patients with PFIC5, are deficient for the nuclear FXR, that has a central role in bile acid (BA) synthesis and homeostasis. Since FXR signaling is also needed for the expression of both ABCB4 and ABCB11, its deficiency results in lack of expression of these two transporters resulting in intracellular BA accumulation as seen in PFIC1 and 2 [69]. Recently, PFIC6 has been described to be caused by bi-allelic missense mutations in *MYO5B*. This protein plays a central role in intracellular transport of membrane proteins and these MYO5B mutants lead to ABCB11 mislocalization and, as a consequence, results in intracellular accumulation of bile salts [7,9]. Based on the pathophysiology of these three disease types, only integrating gene therapy strategies seem suited as possible treatment because the survival benefit of corrected hepatocytes may lead to liver repopulation and subsequent correction of the disease.

Animal models are essential to investigate the feasibility by showing proof of concept of gene therapy for these types of PFIC. For PFIC4, a TJP2 deficient model is needed. The whole body knock-out has been generated but appeared to be lethal during the embryonal period [11]. Therefore additional models, such as a hepatocyte specific knock-out by crossing a *TJP2* gene floxed mouse with an Alb-*Cre* mouse or even a conditionally inducible model need to be generated [70]. FXR-deficient mice have been generated, but in addition to a liver phenotype, this model displays a wide array of symptoms in agreement with the central role of this nuclear receptor in many processes. In addition to impaired liver regeneration, increased hepatic tumorigenesis and cholestasis, intestinal pathology, atherosclerosis and neurological malfunction is seen [71,72,73,74]. This suggests that gene therapy that only targets the liver may be partly therapeutic. Mouse models for MYO5B-deficiency have been generated and the predominant symptom in this model is Microvillus Inclusion Disease (MID). A recent study shows that the MID mouse, having total body knock-out of MYO5B, is not suitable to model cholestasis. The aberrant protein trafficking to the apical membrane of hepatocytes, resulting in the PFIC6 phenotype, is caused by missense mutations affecting the motor domain but not by complete MYO5B-deficiency [75]. The presence of wildtype MYO5B partially corrects the mislocalization of apical proteins in a hepatoma cell line, suggesting gene addition therapy seems a feasible approach. Proof of concept for this strategy would require a new mouse model expressing one of the specific missense mutations identified in PFIC6 patients.

The genome of wild-type AAV consist of a 4.8 Kb long single stranded DNA chain. Although AAV vectors can package a somewhat longer genome, longer genomes result in less efficient packaging or packaging of partial constructs. The maximal capacity that allows efficient packaging is limited to 5.2 Kb [76]. The coding sequences for the proteins deficient in the different types of PFIC are 3753 bp for hATP8B1 (PFIC1), 3963 bp for hABCB11 (PFIC2), 3858 bp for hABCB4 (PFIC3), 3570 bp for hTJP2 (PFIC4), and 1458 bp for hFXR (PFIC5). In PFIC6, the canonical spliced MYO5B variant consist of 5544 bp, but smaller splice variants consist of 2889 and of 1257 bp. As only specific missense mutations in the motor domain of MYO5B cause cholestasis, gene addition therapy of such a smaller splice variant, if functional, may in theory be an option, but only in combination with deleting the expression of the endogenous mutated MYO5B protein. The MYO5B domain binding Rab11 plays a crucial role in the mislocalization of apical proteins in hepatocytes resulting in PFIC6 [75]. This suggests that using AAV-mediated gene therapy to knock-out the expression of this domain using for instance CRISPR/Cas could be an option. Such a treatment would not require homologous repair nor the delivery of a donor template, that both limit the efficacy of in vivo gene correction therapy. Importantly, besides the transgene, AAV constructs consist of one poly A consensus sequence, a promoter, when using a non-integrating strategy, or two homologous arms, in the case of an integrating approach, and the inverted terminal repeats needed for packaging. These requirements make the development of both integrating and non-integrating therapeutic constructs within the limited packaging capacity of AAV challenging, but feasible for PFIC1 to 5. The absence of intrahepatic cholestasis in patients with complete MYO5B deficiency, suggest that a gene therapy that blocks the expression of MYO5B with motor domain missense mutations may partly correct the mislocalization of apical proteins and in theory could be a feasible approach to treat PFIC6.

### 2.4. Prospects of Ex Vivo Gene Therapy for PFIC

Applying gene therapy to isolated patient cells has major important advantages over in vivo approaches, like absence of interference of pre-existing immunity, the lack of an immune response towards the vector, no vector toxicity, exclusive targeting of the affected cell type, and the less invasive procedure [28]. Ex vivo gene therapy was the first form to show therapeutic efficacy in correcting inherited diseases of the hematopoietic stem cells (HSCs) [77]. This ex vivo correction benefited largely from the extensive experience with bone-marrow transplantations for tissue culture and pre-conditioning of the patient. Several HSC ex vivo gene therapies have shown long-term therapeutic efficacy and one has been registered, namely Strimvelis [78,79,80]. Transplantation of donor derived hepatocytes has been applied in patients suffering from different forms of inherited liver disorders [81]. Several studies report a partial correction of the disease indicating functionality of the transplanted hepatocytes. In all studies, this effect appeared to be transient, most likely due to immune responses towards the donor derived hepatocytes. Correcting patient derived hepatocytes would, by overcoming immune mediated loss of transplanted hepatocytes, provide long term correction. However, a small clinical study to treat familial hypercholesterolemia caused by low-density lipoprotein receptor (LDLR) deficiency did not provide therapeutic efficacy [82]. In part, this low efficacy could be explained by the use of a retroviral vector that, in contrast to a lentiviral vector, does not transduce non-dividing cells, such as mature hepatocytes. In addition, this trial also showed the complexity of the procedure including partial resection of the liver to isolate, culture and transduce the amount of hepatocytes needed for therapeutic efficacy. Further, in contrast to bone marrow transplantation, the experience with conditioning of the recipient is limited. All taken together, this approach only renders feasibility if corrected hepatocytes have a survival advantage resulting in a partial repopulation of the liver by the corrected cells. The liver damage observed in all types of PFIC does indicate that corrected hepatocytes may be able to repopulate the liver in these patients. Studies in relevant pre-clinical models for PFIC2 and 3 demonstrate that transplanted hepatocytes indeed repopulate the liver [52,67]. For the other types of PFIC a significant survival advantage of corrected cells has not been demonstrated, hence it is less clear if this approach will be suitable.

Correction of patient hepatocytes using ex vivo gene therapy and subsequent transplantation does require integration of the therapeutic construct in, or correction of, the host genome. The most effective vector for this type of gene therapy are the lentiviral vectors that transduce non-dividing cells such as hepatocytes. These vectors do integrate mostly in active genes which could increase the risk of tumorigenesis. For traditional retroviral vectors this risk indeed resulted in the development of acute leukemia, as shown in two trials [47,83]. In comparison to the vector used in these older studies, the safety of lentiviral vectors has been improved [84]. These third generation lentiviral vectors have been applied in several clinical trials and no tumor formation has been seen [85,86,87]. Ex vivo gene therapy using mature hepatocytes obtained from the patient liver will be a very challenging procedure especially in view of the large number of cells needed. To render this method applicable, cell proliferation will be essential to provide sufficient numbers for effective gene therapy. In this respect the use of stem cells, either induced pluripotent stem cells (iPSCs) or from the liver, remains necessary. In combination with a survival effect, these ex vivo corrected cells can be effective. A recent study for a severe skin disease resulted in complete recovery upon transplanting corrected skin stem cells [88]. For the liver this will be more complicated, but may be possible in future perspective.

### 2.5. Clinical Feasibility

To summarize, in vivo gene therapy using AAV vectors shows safety and efficacy in the clinic for several inherited diseases, including disorders affecting the liver. Within these therapies, the non-integrating approach has been extensively explored. In view of the early onset of severe damage and the persistent proliferation in case of insufficient efficacy, for all PFIC, integrating approaches are necessary for improved clinical application. Ex vivo gene therapy in adult hepatocytes followed by transplantation seems a potential option due to the survival benefit of the corrected cells that will promote liver repopulation. In view of the complicated procedure and inefficient grafting, in vivo gene therapy currently looks more promising for all types of PFIC.

The predominant concern for integrating gene therapy is induction of tumorigenesis, fueled by the early trials in severe combined immunodeficiency (SCID) patients [77]. The current adequate focus on tumorigenesis within gene therapy research will accelerate the development of safe, genome integrating gene therapeutics. State of the art integrating in vivo gene therapy approaches, including CRISPR/Cas9 show great promise in pre-clinical research because integration is guided, and therefore controlled, by Cas9 [56]. Currently, a first AAV-directed Cas9 driven gene therapy clinical trial for Leber’s congenital amaurosis 10 (LCA10) has been approved and is recruiting (NCT03872479). Particularly safety assessments will determine conclusive outcomes for future in vivo application of integrating gene therapies in other inherited diseases.

Easily accessible organs like the eye, cervix, and liver are good targets for integrating gene therapy and future perspectives likely include new therapeutics for disorders within these target organs [89]. Proliferation advantages of corrected hepatocytes in stressed and diseased livers represent an important argument to choose for integrating gene therapy in certain types of PFIC.

Feasibility of gene therapy as a curative treatment differs between the types of PFIC. Deficiency in ABCB11 (PFIC2) show a complicated clinical expectation as the disease is cell autonomous and patients can develop hepatocellular carcinomas (HCC) or cholangiocarcinoma’s (CCC) [90,91,92]. Although integrating gene therapy results in liver repopulation, presence of non-corrected cells cannot be excluded and maintains a permanent risk. Therefore even upon effective treatment in these patients, the risk of developing HCC and subsequent OLT remains [93]. Current available gene therapy methods seem an effective option to prevent liver failure until a suitable donor liver is available, but cannot replace liver transplantation in PFIC2 patients.

In PFIC5, deficiency of FXR, the potential role of gene therapy is comparable to that in patients with PFIC2. FXR is essential for transcription of the *ABCB11* gene and the expression of BSEP in all hepatocytes. Like PFIC2 patients, PFIC5 patients are at risk to develop HCC at an early age. Even upon effective gene therapy, presence of non-corrected hepatocytes results in a persisting risk for HCC development. In addition, the broad range of processes regulated by FXR results in a complicated disease and therefore correction of the diseased liver will not cure the pathology in all affected tissues. PFIC6 is caused by mutations in specific parts of the MYO5B protein, affecting its translocational capacity of proteins to the apical membrane [75]. Most MYO5B-deficient patients dominantly suffer from Microvillus Inclusion Disease [7]. Only for a sub-group of patients suffering from PFIC6, in which the liver pathology is the main symptom, liver directed gene therapy deleting the expression of the mutated MYO5B seems a feasible option.

In view of currently available methods, gene therapy to treat PFIC1, 3, and 4 seems the most feasible for clinical translation. Deficiencies of ABCB4 is the best studied type where three different pre-clinical studies all showed efficacy and proof of concept for liver directed in vivo gene therapy. The liver pathology caused by ATP8B1 deficiency (PFIC1) is also an attractive target for integrating gene therapy since the survival benefit of the corrected cells will promote liver repopulation. However, this approach will not address the extra-hepatic symptoms and it is not clear if an effective treatment will result in the development of a fatty liver, as seen upon OLT in these PFIC patients. In patients suffering from PFIC4, re-expression of TJP2 needed for the formation of functional tight junction formation in the liver, may also result in a survival benefit of the corrected cells [11]. Since pathology caused by TJP2 deficiency seems restricted to the liver, this appears a good target for in vivo gene therapy. Proof of concept studies require the development of a conditional liver specific TJP2 knock-out model for PFIC4 because the whole body knock-out is not viable.

## 3. Conclusions

In this review, we discussed the most recent in vivo and ex vivo approaches for using gene therapy in patients with PFIC, and addressed future developments within this field. Further, we elaborated on the clinical feasibility for all types of PFIC. The safety and efficacy results from many clinical trials conducting gene therapy are sufficiently positive to push the application of gene therapy for PFIC patients closer to the clinic. We can conclude that state of the art in vivo non-integrating gene therapy approaches will most likely not provide lifelong correction, due to a loss of AAV vector genomes caused by hepatocyte proliferation. However, the transient correction can slow down the decrease in liver function. Only integrating strategies that provide survival benefit to the corrected cells seem suitable for sustained efficacy in PFIC.

The feasibility to translate state of the art in vivo integrating gene therapies, upon showing proof of concept in pre-clinical models, into the clinic depends on the type of PFIC. Although integrating gene therapy would cure the PFIC2 and 5 phenotype, these patients remain at risk to develop HCC or CCC. Gene therapy for PFIC6 will only be effective for the small group of patients with the mutations in the motor domain of MYO5B that dominantly suffer from liver symptoms. Although MYO5B is too large to be packaged into AAVs, treating PFIC6 by knocking out gene expression of the mutated protein in the liver using AAV-mediated gene therapy or another approach to deliver CRISPR/Cas9 specifically to the hepatocytes, in theory could be a promising option. In most of MYO5B deficient patients, the pathology in the intestine is much more severe and will not be mitigated by AAV-mediated in vivo liver-directed gene therapy. PFIC1, 3 and 4 do seem good candidates for integrating gene therapy strategies targeting the diseased hepatocytes and could provide life-long correction in patients suffering from these severe life-threatening disorders.
